# Whole-exome sequencing expands the roles of novel mutations of organic anion transporting polypeptide, ATP-binding cassette transporter, and receptor genes in intrahepatic cholestasis of pregnancy

**DOI:** 10.3389/fgene.2022.941027

**Published:** 2022-08-15

**Authors:** Xianxian Liu, Jiusheng Zheng, Siming Xin, Yang Zeng, Xiaoying Wu, Xiaoming Zeng, Hua Lai, Yang Zou

**Affiliations:** Key Laboratory of Women’s Reproductive Health of Jiangxi Province, Jiangxi Provincial Maternal and Child Health Hospital, Nanchang, Jiangxi, China

**Keywords:** ICP, WES, OTAPs, ABCs, receptors, novel mutations

## Abstract

**Background:** Intrahepatic cholestasis of pregnancy (ICP) is associated with a high incidence of fetal morbidity and mortality. Therefore, revealing the mechanisms involved in ICP and its association with fetal complications is very important.

**Methods:** Here, we used a whole-exome sequencing (WES) approach to detect novel mutations of organic anion transporting polypeptide (OTAP) genes, ATP-binding cassette transporter (ABC) genes, and receptor genes associated with ICP in 249 individuals and 1,029 local control individuals. Two available tools, SIFT and PolyPhen-2, were used to predict protein damage. Protein structuremodeling and comparison between the reference and modified protein structures were conducted by SWISS-MODEL and Chimera 1.14rc software, respectively.

**Results:** A total of 5,583 mutations were identified in 82 genes related to bile acid transporters and receptors, of which 62 were novel mutations. These novel mutations were absent in the 1,029 control individuals and three databases, including the 1,000 Genome Project (1000G_ALL), Exome Aggregation Consortium (ExAC), and Single-Nucleotide Polymorphism Database (dbSNP). We classified the 62 novel loci into two groups (damaging and probably damaging) according to the results of SIFT and PolyPhen-2. Out of the 62 novel mutations, 24 were detected in the damaging group. Of these, five novel possibly pathogenic variants were identified that were located in known functional genes, including *ABCB4* (Ile377Asn), *ABCB11* (Ala588Pro), *ABCC2* (Ile681Lys and Met688Thr), and *NR1H4* (Tyr149Ter). Moreover, compared to the wild-type protein structure, *ABCC2* Ile681Lys and Met688Thr protein structures showed a slight change in the chemical bond lengths of ATP-ligand binding amino acid side chains. The combined 32 clinical data points indicate that the mutation group had a significantly (*p* = 0.04) lower level of Cl ions than the wild-type group. Particularly, patients with the 24 novel mutations had higher average values of alanine transaminase (ALT), aspartate transaminase (AST), alkaline phosphatase (ALP), total bile acids (TBA), high-density lipoprotein (HDL), and low-density lipoprotein (LDL) than patients with the 38 novel mutations in the probably damaging group and the local control individuals.

**Conclusion:** The present study provides new insights into the genetic architecture of ICP involving these novel mutations.

## Introduction

Intrahepatic cholestasis of pregnancy (ICP) is the most common pregnancy-specific liver disease, and it is characterized by skin pruritus, predominantly on the palms and soles and elevated levels of total serum bile acids (≥10 μmol/L) and liver enzymes (Ovadia and Williamson, 2016). This disease typically commences in the late second or third trimester of pregnancy and resolves rapidly after delivery in the early postpartum period (Rook et al., 2012; Puljic et al., 2015). The incidence of ICP was reported to range from 0.2 to 15.6% depending on ethnicities and geographic locations (Stulic et al., 2019). In particular, the incidence rate is higher in the Scandinavian, Bolivian, and Chilean populations (Reyes et al., 1979; Saleh and Abdo, 2007; Rook et al., 2012; Puljic et al., 2015; Simjak et al., 2015). The recurrence rate of ICP during the next pregnancy can be as high as 40–60% (Ovadia and Williamson, 2016).

ICP is linked with a higher risk of spontaneous preterm birth, fetal asphyxia, meconium-stained amniotic fluid, low Apgar scores, and fetal death (Glantz et al., 2004; Geenes et al., 2014; Williamson and Geenes, 2014). Many researchers found that pregnant women with serum bile acid levels of 100 μmol/L or more have a significantly increased risk of stillbirth (Kawakita et al., 2015; Ovadia et al., 2019). In addition, ICP is associated with a disrupted metabolic profile and causes metabolic disease. Thus, it has long-term consequences for the health of the mother and child (Papacleovoulou et al., 2013; Wikstrom Shemer et al., 2015). Therefore, understanding the underlying genetic factors of ICP disease is of great significance to patient diagnosis and treatment.

The etiology of ICP is complex and not fully understood. The pathogenesis of ICP is multifactorial and is related to the interactions among genetic, hormonal, immunological, and environmental factors (Sampson, 1927; Xiao et al., 2021). The familial clustering analysis in pedigree studies showed a high incidence of these factors in the mothers and sisters of patients with ICP. There are population-specific risk differences and a high recurrence rate in these individuals, which might indicate the importance of genetic components in ICP (Reyes et al., 1976; Eloranta et al., 2001; Dixon and William, 2008). Previous studies have demonstrated that OTAP genes, ABC genes, and receptor genes play a pivotal role in the control of bile acid homeostasis (Van Mil et al., 2007; Kakizaki et al., 2011; Yan et al., 2015; Ahmad and Haeusler, 2019; Turro et al., 2020). Yan et al., 2015 confirmed the role of *SLCO1B3* in bile acid transport. Ontsouka et al., 2021 reported that placental *SLC10A2*, *SLCO4A1*, and *ABCC2* mRNA levels were positively correlated with bile acid concentrations in ICP patients (). Therefore, considering that women with ICP exhibited elevated serum bile acid levels and that OTAP abnormalities can result in altered bile acid levels, we speculated that mutations in OTAP genes might be present in women with ICP. In addition, mutations in *ABCB4*, *ABCB11*, and *FXR* were confirmed to be associated with ICP disease (Van Mil et al., 2007; Castano et al., 2010; Dixon et al., 2014; Dixon et al., 2017; Turro et al., 2020; Wolski et al., 2020). To the best of our knowledge, until now, most studies have mainly focused on functionally known genes, such as *ABCB4*, *ABCB11*, *ABCC2*, *FXR*, *ATP8B1*, and *TJP2*, that confer susceptibility to ICP (Dixon et al., 2014; Dixon et al., 2017). However, the roles of other bile acid receptor genes, such as *CAR*, *AhR*, *VDR*, and *PXR*, seem to be less studied. Accumulating evidence suggests that the role of these receptor genes as promising drug targets in cholestasis is now emerging since they play a crucial role in the regulation of bile acid synthesis, detoxification, and transport (Ahmad and Haeusler, 2019). However, to date, only a few receptor mutations associated with ICP disease have been identified (Van Mil et al., 2007; Castano et al., 2010; Lai et al., 2022). Therefore, it is necessary to systematically identify ICP-associated bile acid transporter and receptor gene susceptibility mutations.

In past decades, most of the genetic mutations have been found in families or sequenced in a limited number of individuals with sporadic ICP, and this makes the identification of rare variants challenging. However, the 1000 Genomes Project revealed that rare variants constitute the majority of polymorphic sites in human populations and have higher effect sizes on human diseases than common variants (Genomes Project et al., 2012; Wang et al., 2021). Fortunately, by taking advantage of high-throughput sequencing in a large population, whole-genome sequencing (WGS)/WES has been shown to be a useful and efficient tool for identifying potentially pathogenic rare mutations in rare diseases, such as ICP (Turro et al., 2020). Previously, we performed WES to identify the novel gene *ANO8* as a gene risk factor for ICP and novel mutations in ABC transporter genes that were associated with ICP disease in 151 ICP samples (Liu et al., 2020; Liu et al., 2021). We would like to further conduct analyses of comprehensive sequencing of the bile acid transporter and receptor gene series in a larger cohort of ICP cases.

Given the aforementioned background, we aimed to systematically identify mutations associated with bile acid transporters and receptor genes in 249 ICP individuals. Our particular focus will be on new functional mutations and their relationship with clinical data and pregnancy outcomes in this study.

## Materials and methods

### Patients and clinical data

We recruited a total of 249 pregnant women who did not have other liver diseases and were diagnosed with ICP disease between 2018 and 2022. These peripheral blood samples were collected from the Department of Obstetrics, Jiangxi Provincial Maternal and Child Health Hospital in Nanchang, China. Additionally, thirty-five available clinical characteristics, including basic patient information, were recorded. This basic information included the age at diagnosis, body mass index (BMI), gestational age, gravidity, and parity. It also included the four main serum biochemical indexes of maternal and neonatal data, such as the ion concentrations of K, Na, Cl, Ca, P, and Mg. The basic information also included routine blood tests, such as white blood cell (WBC), red blood cell (RBC), and platelet (PLT) counts and red blood cell distribution width SD (RDW-SD), and liver function indexes, including ALT, AST, γ-glutamyl transpeptidase (GGT), ALP, TBA, cholyglycine (CG), total bilirubin (TBIL), direct bilirubin (DBIL), and indirect bilirubin (IDBIL) levels. Additionally, it included lipid-related indices, including total cholesterol (CHOL), triglyceride (TG), HDL, LDL, and uric acid (UA) levels, and outcomes of pregnant women and newborn babies, including newborn birth weight, Apgar score, bleeding amount, preterm birth, meconium staining amniotic fluid (MSAF), and intrauterine fetal death. The measurement of these blood biochemical variables can be found in our previous studies (Liu et al., 2020; Liu et al., 2021). In brief, the routine blood tests were determined by a Sysmex-xn-2000 automated blood cell analyzer (Sysmex Corporation, Japan). The other serum biochemical indices were examined by an AU5800 automatic biochemical analyzer (Beckman Coulter, Inc., United States). A summary of the statistics for all the clinical features in 249 samples is shown in Table 1. The clinical data from 151 of these samples were described in our previous study (Liu et al., 2020; Liu et al., 2021). Each participating woman gave written informed consent.

**TABLE 1:** Descriptive statistics of thirty-five clinical characteristics of 249 ICP patients.

Characteristics	N	Mean	SD^a^	Min	Max
**Basic information**					
Age (years)	245	29.22	5.08	17	43
BMI (kg/m^2^)	239	25.80	3.38	17.08	38.50
Gestational age (days)	228	263.43	15.32	207	290
Gravidity (Times)	244	2.39	1.50	1	8
Parity (Times)	244	0.66	0.78	0	4
**Serum biochemical index**					
K (mmol/L)	249	4.00	0.34	3.20	6.40
Na (mmol/L)	249	137.27	2.24	132.00	145.00
Cl (mmol/L)	249	103.96	2.53	97.00	112.00
Ca (mmol/L)	249	2.37	0.17	2.00	2.90
P (mmol/L)	249	1.18	0.21	0.70	1.80
Mg (mmol/L)	248	0.80	0.14	0.20	1.89
WBC (×10^9^)	249	8.57	2.70	4.11	24.23
RBC (×10^9^)	249	3.81	0.42	2.65	5.52
PLT (×10^9^)	249	198.14	62.13	75.00	476.00
RDW-SD (fL)	249	46.03	4.88	36.20	67.30
ALT (U/L)	249	100.12	126.09	3.00	595.00
AST (U/L)	249	84.79	96.52	12.00	509.00
GGT (U/L)	244	30.68	38.10	3.00	359.00
ALP (U/L)	244	173.82	79.19	39.00	487.00
TBA (μmol/L)	248	42.10	38.63	4.20	286.80
CG (mg/L)	198	11.06	14.07	0.30	88.60
TBIL (μmol/L)	245	14.57	7.67	5.30	67.90
DBIL (μmol/L)	245	6.77	6.10	0.90	52.50
IDBIL (μmol/L)	245	7.81	3.32	2.10	26.90
CHOL (mmol/L)	241	6.35	1.44	3.16	13.25
TG (mmol/L)	241	3.51	1.50	1.20	11.10
HDL (mmol/L)	241	1.96	0.52	0.92	5.34
LDL (mmol/L)	241	2.97	1.32	0.04	7.34
UA (μmol/L)	246	333.37	94.50	111.00	701.00
**Outcomes of pregnant women and newborn baby**					
Birth weight (kg)	225	3.04	0.56	1.23	5.30
Apgar score (1–10)	219	9.36	0.69	6.00	10.00
Bleeding count (ml)	221	263.14	94.95	80.00	810.00
Preterm birth	64	-	-	-	-
MSAF	51	-	-	-	-
Intrauterine fetal death	0	-	-	-	-

a Standard deviation.

### Whole-exome sequencing

A total of 249 genomic DNA samples were extracted from peripheral blood by an Axy Prep Blood Genomic DNA Mini Prep Kit (Item No. 05119KC3, Axygen Scientific, Inc., Union City, CA, United States) and dissolved in Tris-EDTA buffer. The quality and concentrations of the DNA samples were determined by a Nanodrop-1000 spectrophotometer (Thermo Fisher, United States). The integrity of the DNA samples was examined by gel electrophoresis. After the quality control analysis, a total of 249 qualified DNA samples were subjected to exome sequencing following the standard manufacturer’s protocol. The detailed procedures were conducted as previously described (Liu et al., 2020; Liu et al., 2021). Briefly, 1 µg of genomic DNA was randomly sheared into short fragments (150–250 bp) using Covaris technology (Woburn MA). The prepared DNA fragments were amplified by ligation-mediated PCR, purified, and hybridized to the BGI Exon Kit V4 (BGI, Shenzhen, China) for enrichment. The enriched library was then loaded on BGISEQ-500 (BGI, Shenzhen, China) platforms and subjected to high-throughput sequencing. Additionally, whole-exome sequencing was also conducted in 1,029 control individuals. These control samples without ICP or liver disease were recruited as negative control individuals in the same periods. We collected these local control individuals to compare the candidate locus frequency differences between cases and local control individuals.

### Variant calling, annotations, filtering, and prioritization

The bioinformatics analysis was also previously described (Liu et al., 2020; Liu et al., 2021). In the aggregate, reads from the BGISEQ-500 machine containing sequencing adapters and low-quality reads (read depth <15 and genotype quality score <20) were removed. The clean reads were aligned to the human reference genome (UCSC Genome Browser GRCh37/hg19) using Burrows–Wheeler Aligner (BWA) software (Li and Durbin, 2009). Then, variant calls and annotations were conducted by the genome Analysis Toolkit (GATK) and ANNOVAR tool (Mckenna et al., 2010; Wang et al., 2010), respectively. After that, we removed variants with a minor allele frequency (MAF) > 0.05 in the 1,029 control individuals, 1000G_ALL (http://www.internationalgenome.org/), ExAC (http://exac.broadinstitute.org/), and dbSNP (https://www.ncbi.nlm.nih.gov/snp/) databases. Then, variants, such as missense, nonsense, loss or gain of function mutations of OTAP genes, ABC genes, and receptor genes, were included in the subsequent analysis. In particular, we concentrated on novel variants that were filtered by National Center for Biotechnology Information (NCBI) and Ensembl. Additionally, SIFT and PolyPhen-2 tools were applied to annotate and predict the functions of the mutation variants. These were prioritized with attention given to novel variants that would likely have functional effects. For instance, a variant was highlighted when it was a loss- or gain-of-function variant or predicted to be simultaneously deleterious by SIFT and PolyPhen-2 software. According to the prediction results, predictions were defined as damaging when the two prediction software results both reached the damaging level. The other variants were assigned to the probably damaging group.

### Statistical analysis

We performed the *sapply* function to calculate descriptive statistics for 35 clinical data points. The *t* test method was performed to analyze the potential significant differences between gene mutations and wild-type genotypes for clinical characteristics. The result was considered significant when the *p* value was below 0.05. Fisher’s test was used to test the significance of the frequencies of the 249 ICP patients and 1,029 control individuals. In addition, comparisons of the average value differences in six clinical parameters, including ALT, AST, ALP, TBA, HDL, and LDL, among the three groups were performed by one-way ANOVA. The *pie* function was used to draw the percentages of the types of OTAP, ABC, and receptor gene mutations. Logistic regression analysis was performed to assess the relationship between the clinical parameters (age, gestational age, BMI, gravidity, and parity) and the mutations. All the aforementioned analyses were performed by R software.

### Sanger sequencing

To validate putative mutations, Sanger sequencing was conducted. We selected 12 interesting novel mutations from WES analyses to be confirmed. The twelve mutations were selected for sequencing mainly based on functional prediction results (damaging group) and the classification of the members of the OTAP, ABC, and receptor gene superfamilies. The primers flanking the 12 candidate loci were designed based on the reference genome of the human genome from GenBank in NCBI using Primer Premier 5 software. The details of the PCR primers, their optimum annealing temperatures, and the fragment lengths of the amplified products are shown in Supplementary Table S1.

### Evolutionary conservation analysis

The evolutionary conservation analysis of the amino acids encoded by the new functional sites in OTAP genes, ABC genes, and receptor genes in the damaging group was performed among vertebrates, including gibbons, gorillas, goats, macaques, mice, cats, cows, horses, pigs, and sheep, using genomic alignments of the Ensembl Genome Browser.

### Protein structure modeling

There are two steps to complete the protein structure modeling. First, the reference and modified (*ABCC2* Ile681Lys and Met688Thr) protein sequences were submitted to SWISS-MODEL (https://swissmodel.expasy.org/) to build the model. Then, the reference and modified protein models were compared simultaneously using the Chimera 1.14rc package.

## Results

### Clinical presentation

During the study period, 249 cases of ICP were diagnosed. Of these 249 women, fourteen women (5.62%, 14/249) had >1 ICP-affected pregnancy. Among them, two had a history of ICP stillbirths. One and 51 pregnant women presented with fetal distress and MSAF, respectively. In addition, 227 women of the 249 sampled women delivered their babies. Out of the 227 women, 173 individuals (76.21%, 173/227) gave birth by cesarean section, 53 (23.35%, 53/227) gave birth by vaginal delivery, and one (0.44%, 1/227) gave birth by vaginal delivery with forceps. Sixty-four (28.44%, 64/225) babies were born prematurely, and 32 (14.22%, 32/225) babies weighed less than 2.5 kg. No indications of fetal death were observed *in utero*.

### Whole-exome sequencing results of the variants of organic anion transporting polypeptide, ATP-binding cassette transporter, and receptor genes in 249 intrahepatic cholestasis of pregnancy samples

**FIGURE 1:**
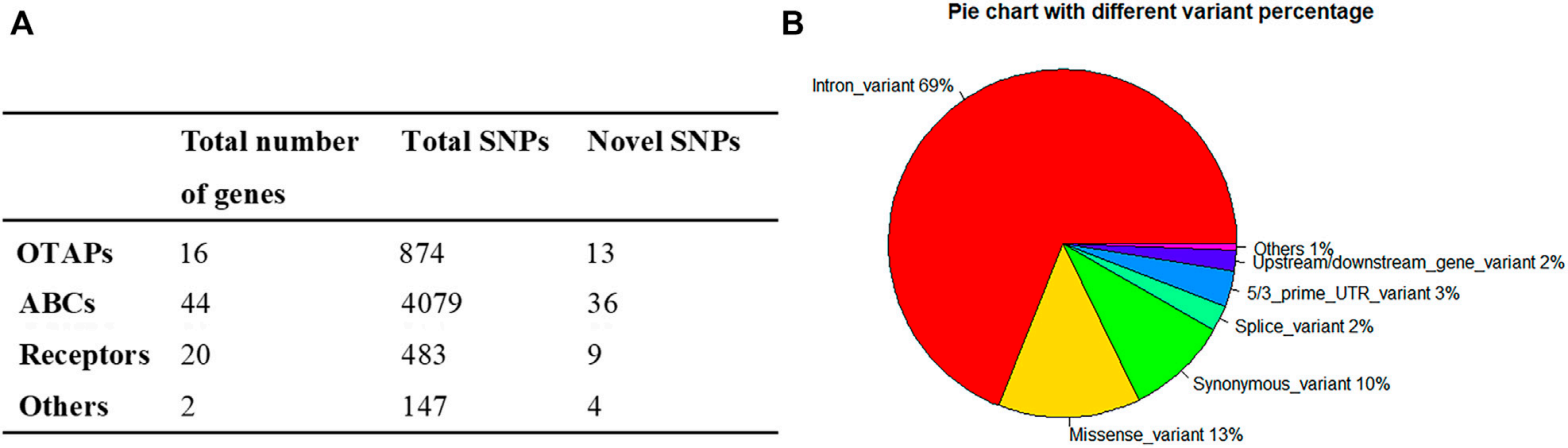
The distribution and numbers of genes and genetic variants from the WES data for ICP. **(A)** The total number of genes, identified SNPs, and novel SNPs in OTAP genes, ABC genes, receptor genes, and other genes are summarized. **(B)** The percentage of the types of genetic variants in the four gene series.

To survey the novel possible pathogenic variants of OTAP genes, ABC genes, and receptor genes in ICP, we sequenced whole exomes from 249 individuals in the study cohort. In total, we identified 5,583 genetic variants in 82 genes (Supplementary Table S2) that were associated with bile acid transporters and receptors. These included 874 variants in 16 OTAP genes, 4,079 variants in 44 ABC genes, 483 variants in 20 receptors, and 147 variants in the *ATP8B1* and *TJP2* genes (Figure 1A). These types of variants included 3,854 intron variants, 735 missense variants, 531 synonymous variants, 185 5/3 prime UTR variants, 135 splice variants, 106 upstream/downstream gene variants, and 29 start lost/stop gained variants, and 8 structural interaction variants. The percentage of these types of variants is shown in Figure 1B. When MAF was controlled at 0.05, a total of 449 variants were conserved for further analysis (Supplementary Table S3, Table 2). Out of these 449 variants, 62 were novel variants. These included 13 novel variants in OTAP genes, 36 novel variants in ABC genes, 9 novel variants in receptor genes, and 2 novel variants in the *ATP8B1* and *TJP2* genes (Table 2).

**TABLE 2:** Sixty-two novel mutations were identified in 249 Han Chinese patients with ICP disease.

Order	Gene	Patients	Chr	Position	Codon change	Protein change	SIFT^a^	PolyPhen2^b^	Frequencies in controls	Frequencies in 249 ICP	*p* value^c^ (controls-ICP)
1	*SLC10A1*	ICP133,135	chr14	70,252,871	tgC/tgA	Cys170Ter	-	-	0/1,029	10.44% (26/249)	*p* < 2.2e-16
2	*SLC10A1*	ICP147	chr14	70,263,859	aAc/aGc	Asn5Ser	0.035 (D)	0.501 (P)			
3	*SLC12A3*	ICP258	chr16	56,938,333	gaG/gaT	Glu970Asp	0.0 (D)	0.998 (D)			
4	*SLCO1A2*	ICP187	chr12	21,459,915	Tat/Cat	Tyr115His	0.001 (D)	0.984 (D)			
5	*SLCO1B1*	ICP31	chr12	21,375,244	Cct/Act	Pro565Thr	0.002 (D)	0.958 (D)			
6	*ABCA2*	ICP234	chr9	139,905,695	Cac/Tac	His1986Tyr	0.003(D)	0.992 (D)			
7	*ABCA2*	ICP212	chr9	139,910,450	aGc/aTc	Ser1124Ile	0.001 (D)	0.876 (P)			
8	*ABCA3*	ICP177	chr16	2,350,016	cAc/cTc	His534Leu	0.004 (D)	0.67 (P)			
9	*ABCA6*	ICP262	chr17	67,096,973	Caa/Taa	Gln993Ter	-	-			
10	*ABCA7*	ICP208	chr19	1,056,910	Gcc/Ccc	Ala1531Pro	0.015 (D)	0.968 (D)			
11	*ABCA10*	ICP220	chr17	67,145,044	gAg/gTg	Glu1519Val	0.0 (D)	0.951 (D)			
12	*ABCA10*	ICP257	chr17	67,150,447	Gga/Aga	Gly1239Arg	0.0 (D)	1.0 (D)			
13	*ABCA12*	ICP207	chr2	215,910,726	cCc/cTc	Pro236Leu	0.004 (D)	0.963 (D)			
14	*ABCA13*	ICP112	chr7	48,354,004	tCa/tGa	Ser3286Ter	-	-			
15	*ABCB4*	ICP192	chr7	87,073,079	aTt/aAt	Ile377Asn	0.0 (D)	0.994 (D)			
16	*ABCB11*	ICP236	chr2	169,826,602	Gct/Cct	Ala588Pro	0.0 (D)	0.999 (D)			
17	*ABCC2*	ICP274	chr10	101,572,849	aTa/aAa	Ile681Lys	0.001 (D)	0.756 (P)			
18	*ABCC2*	ICP236	chr10	101,572,870	aTg/aCg	Met688Thr	0.039 (D)	0.995 (D)			
19	*ABCC6*	ICP260	chr16	16,276,764	gGc/gTc	Gly656Val	0.0 (D)	1.0 (D)			
20	*ABCC11*	ICP256	chr16	48,221,322	aTg/aCg	Met908Thr	0.0 (D)	0.996 (D)			
21	*ABCC11*	ICP224	chr16	48,264,401	tgG/tgA	Trp61Ter	-	-			
22	*ABCG2*	ICP256,257	chr4	89,042,890	Ata/Tta	Ile196Leu	0.004 (D)	0.967 (D)			
23	*CHRM3*	ICP245	chr1	240,072,471	Cag/Tag	Gln574Ter	-	-			
24	*NR1H4*	ICP270	chr12	100,904,893	taT/taA	Tyr149Ter	-	-			
25	*SLC10A2*	ICP32	chr13	103,698,602	Gca/Aca	Ala310Thr	0.431(T)	0.001 (B)	0/1,029	16.47% (41/249)	*p* < 2.2e-16
26	*SLC51B*	ICP227	chr15	65,342,361	Gct/Tct	Ala7Ser	0.242 (T)	0.018 (B)			
27	*SLCO1A2*	ICP227	chr12	21,422,546	gAg/gGg	Glu650Gly	0.092 (T)	0.005 (B)			
28	*SLCO3A1*	ICP197	chr15	92,397,216	aaC/aaA	Asn26Lys	0.118 (T)	0.005 (B)			
29	*SLCO6A1*	ICP199	chr5	101,834,527	Cac/Gac	His8Asp	0.194 (T)	0.013 (B)			
30	*SLCO1B3*	ICP247	chr12	21,008,070	Ctt/Gtt	Leu65Val	0.356 (T)	0.03 (B)			
31	*SLCO1B3*	ICP41	chr12	21,036,512	aCc/aTc	Thr553Ile	0.428 (T)	0.15 (B)			
32	*SLCO4C1*	ICP188	chr5	101,582,963	Atc/Gtc	Ile602Val	0.404 (T)	0.085 (B)			
33	*ABCA4*	ICP32,35,76,159	chr1	94,528,138	gaC/gaA	Asp644Glu	0.426 (T)	0.075 (B)			
34	*ABCA6*	ICP198	chr17	67,097,068	aGa/aTa	Arg961Ile	0.168 (T)	0.285 (B)			
35	*ABCA8*	ICP251	chr17	66,925,785	Ctt/Ttt	Leu286Phe	0.238 (T)	0.132 (B)			
36	*ABCA9*	ICP30	chr17	66,986,059	Att/Gtt	Ile1284Val	0.189 (T)	0.073 (B)			
37	*ABCA9*	ICP141	chr17	67,039,709	Gta/Ata	Val241Ile	0.444 (T)	0.02 (B)			
38	*ABCA13*	ICP171	chr7	48,273,681	aAa/aCa	Lys277Thr	0.001 (D)	0.02 (B)			
39	*ABCA13*	ICP161	chr7	48,311,451	Atg/Gtg	Met730Val	0.064 (T)	0.002 (B)			
40	*ABCB4*	ICP113	chr7	87,035,653	aGt/aAt	Ser1153Asn	0.133 (T)	0.006 (B)			
41	*ABCB4*	ICP113	chr7	87,041,219	Gat/Aat	Asp972Asn	0.658 (T)	0.007 (B)			
42	*ABCB6*	ICP174	chr2	220,083,379	aAc/aGc	Asn6Ser	0.987 (T)	0.002 (B)			
43	*ABCB8*	ICP109	chr7	150,733,247	atC/atG	Ile384Met	0.059 (T)	0.34 (B)			
44	*ABCB9*	ICP169	chr12	123,433,328	cTg/cCg	Leu299Pro	0.09 (T)	0.803 (P)			
45	*ABCC2*	ICP196	chr10	101,564,025	Att/Gtt	Ile487Val	0.179 (T)	0.03 (B)			
46	*ABCC3*	ICP187	chr17	48,734,175	gTg/gGg	Val112Gly	0.09 (T)	0.544 (P)			
47	*ABCC4*	ICP160	chr13	95,899,970	Aga/Gga	Arg38Gly	0.326 (T)	0.214 (B)			
48	*ABCC9*	ICP190	chr12	21,995,312	Gtt/Ctt	Val1137Leu	0.299 (T)	0.006 (B)			
49	*ABCC10*	ICP271	chr6	43,400,107	tCc/tGc	Ser130Cys	0.036 (D)	0.025 (B)			
50	*ABCC10*	ICP254	chr6	43,400,487	Acc/Ccc	Thr257Pro	0.266 (T)	0.162 (B)			
51	*ABCG2*	ICP160	chr4	89,013,480	gGc/gAc	Gly625Asp	0.082(T)	0.327 (B)			
52	*AhR*	ICP224	chr7	17,379,056	aAc/aGc	Asn536Ser	0.059 (T)	0.081 (B)			
53	*AhR*	ICP160	chr7	17,379,445	Cag/Gag	Gln666Glu	0.211 (T)	0.004 (B)			
54	*CHRM2*	ICP201	chr7	136,699,745	Atg/Ctg	Met45Leu	0.259 (T)	0.071 (B)			
55	*NR1H3*	ICP10	chr11	47,281,486	cCc/cTc	Pro69Leu	0.0 (D)	0.003 (B)			
56	*NR1H4*	ICP232	chr12	100,926,356	gAg/gGg	Glu199Gly	0.09 (T)	0.314 (B)			
57	*NR1I2*	ICP32	chr3	119,531,653	Cag/Aag	Gln253Lys	0.526 (T)	0.001 (B)			
58	*NR1I2*	ICP44	chr3	119,536,031	cAg/cGg	Gln465Arg	0.702 (T)	0.003 (B)			
59	*ATP8B1*	ICP61	chr18	55,315,872	Gtg/Ctg	Val1202Leu	0.783 (T)	0.012(B)			
60	*ATP8B1*	ICP240	chr18	55,328,579	aAg/aGg	Lys845Arg	0.525 (T)	0.002(B)			
61	*TJP2*	ICP75	chr9	71,843,001	aAg/aGg	Lys506Arg	0.476 (T)	0.02 (B)			
62	*TJP2*	ICP84	chr9	71,862,985	Gcg/Tcg	Ala940Ser	1.0 (T)	0.705 (P)			

a D: disease-causing, T: tolerated.

b D: probably damaging, P: possible damaging, B: benign.

c The significance of differences in frequencies between 249 ICP, patients, and 1,029 controls.

The italic value means that the significance of differences in frequencies between 249 ICP patients and 1029 controls. P < 0.05, the difference is significant.

These 62 novel mutations were divided into two groups, damaging and probably damaging, according to the prediction results (Table 2). The damaging group had 24 novel mutations, including five in OTAPs, seventeen in ABC genes, and two in receptor genes. For OTAP genes, five novel variants (*SLC10A1* Cys170Ter, Asn5Ser, *SLC12A3* Glu970Asp, *SLCO1A2* Tyr115His, and *SLCO1B1* Pro565Thr) were first detected. Seventeen novel mutations were identified in ABC series genes, including 9 in ABCA genes (*ABCA2* His1986Tyr and Ser1124Ile, *ABCA3* His534Leu, *ABCA6* Gln993Ter, *ABCA7* Ala153Pro, *ABCA10* Glu1519Val, and Gly1239Arg, *ABCA12* Pro236Leu, *ABCA13* Ser3286Ter), 2 in ABCB genes (*ABCB4* Ile377Asn and *ABCB11* Ala588Pro), 5 in ABCC genes (*ABCC2* Ile681Lys and Met688Thr, *ABCC6* Gly656Val, *ABCC11* Met908Thr, and Trp61Ter), and 1 in an ABCG gene (*ABCG2* Ile196Leu). In addition, two mutations in receptor genes (*CHRM3* Gln574 and *NR1H4* Tyr149Ter) were also detected. The frequency of these mutations in the 249 ICP individuals reached 10.44% (26/249). The difference in the frequency of these mutations between the 249 ICP cases and 1,029 control individuals was significant (*p* < 2.2e-16).

In addition, 38 novel mutations were assigned to the probably damaging group, including 8 in OTAP genes (*SLC10A2* Ala310Thr, *SLC51B* Ala7Ser, *SLCO1A2* Glu650Gly, *SLCO3A1* Asn26Lys, *SLCO6A1* His8Asp, *SLCO1B3* Leu65Val and Thr553Ile, and *SLCO4C1* Ile602Val), 7 in ABCA genes (*ABCA4* Asp644Glu, *ABCA6* Arg961Ile, *ABCA8* Leu286Phe, *ABCA9* Ile1284Val, Val241Ile, and *ABCA13* Lys277Thr and Met730Val), 5 in ABCB genes (*ABCB4* Ser1153Asn and Asp972Asn, *ABCB6* Asn6Ser, *ABCB8* Ile384Met, and *ABCB9*Leu299Pro), 6 in ABCC genes (*ABCC2* Ile487Val, *ABCC3* Val112Gly, *ABCC4* Arg38Gly, *ABCC9* Val1137Leu, *ABCC10* Ser130Cys, and Thr257Pro), and 1 in an ABCG gene (*ABCG2* Gly625Asp). These 62 novel mutations were absent in the 1,029 control individuals and the 1000 Genomes Project, ExAC, and dbSNP databases.

### Sanger sequencing to validate novel mutations

**FIGURE 2:**
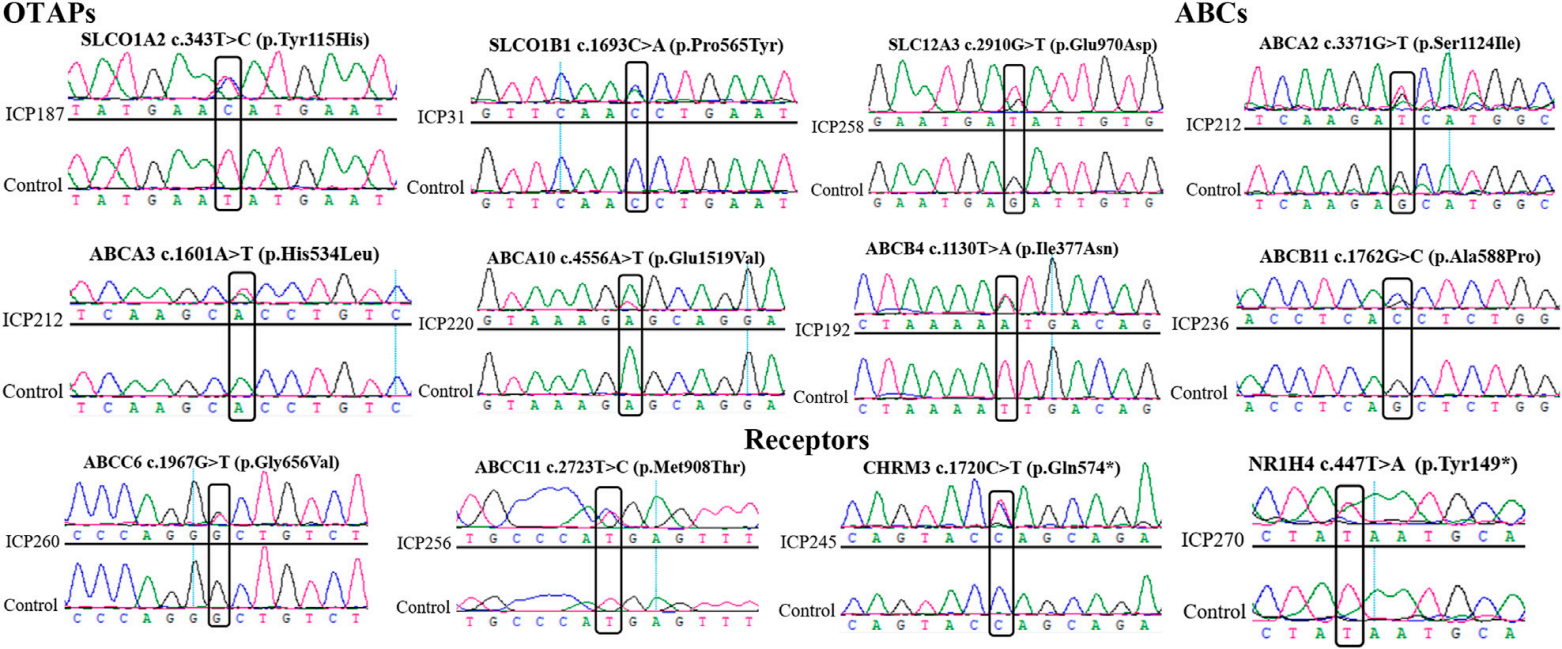
Sanger sequencing to validate the novel mutations in the OTAP genes, ABC genes, and receptor genes. The box represents the mutation site.

Sanger sequencing was used to confirm the 12 possibly pathogenic mutations in the damaging group. The results (Figure 2) were all consistent with WES.

### Evolutionary conservation analysis

**FIGURE 3:**
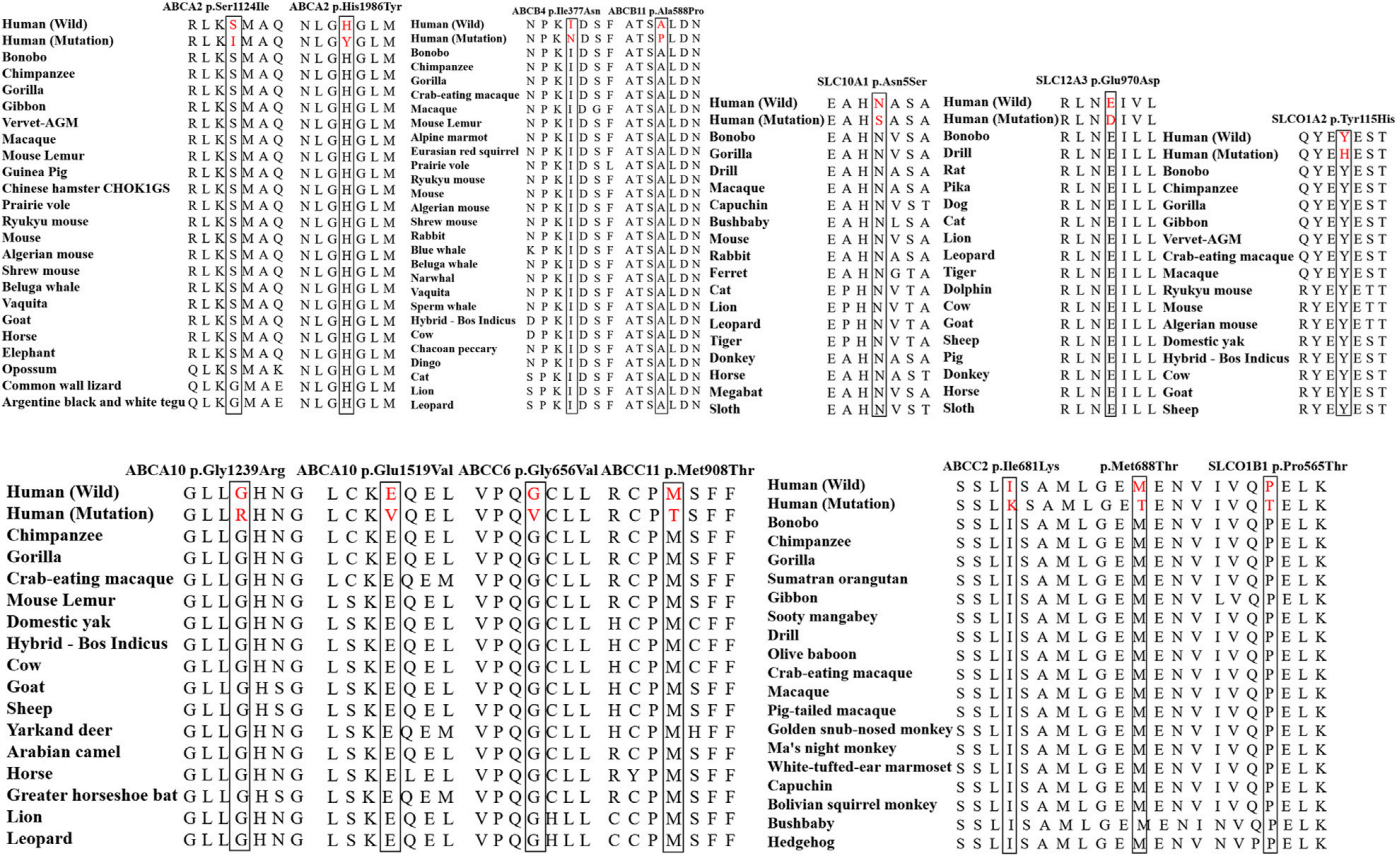
The evolutionary conservation analysis of the mutations in the damaging group among vertebrates, including chimpanzees, gorillas, rats, dogs, pigs, and cows. The amino acids in the red horizontal line were highly conserved.

The Evolutionary conservative analysis results showed that most mutations belonging to the damaging group were highly conserved among vertebrate species, including mice, cats, cows, horses, pigs, and sheep (Figure 3 and Supplementary Figure S1).

### Comparison of the protein structural model of the *ABCC2* Ile681Lys and Met688Thr mutations

**FIGURE 4:**
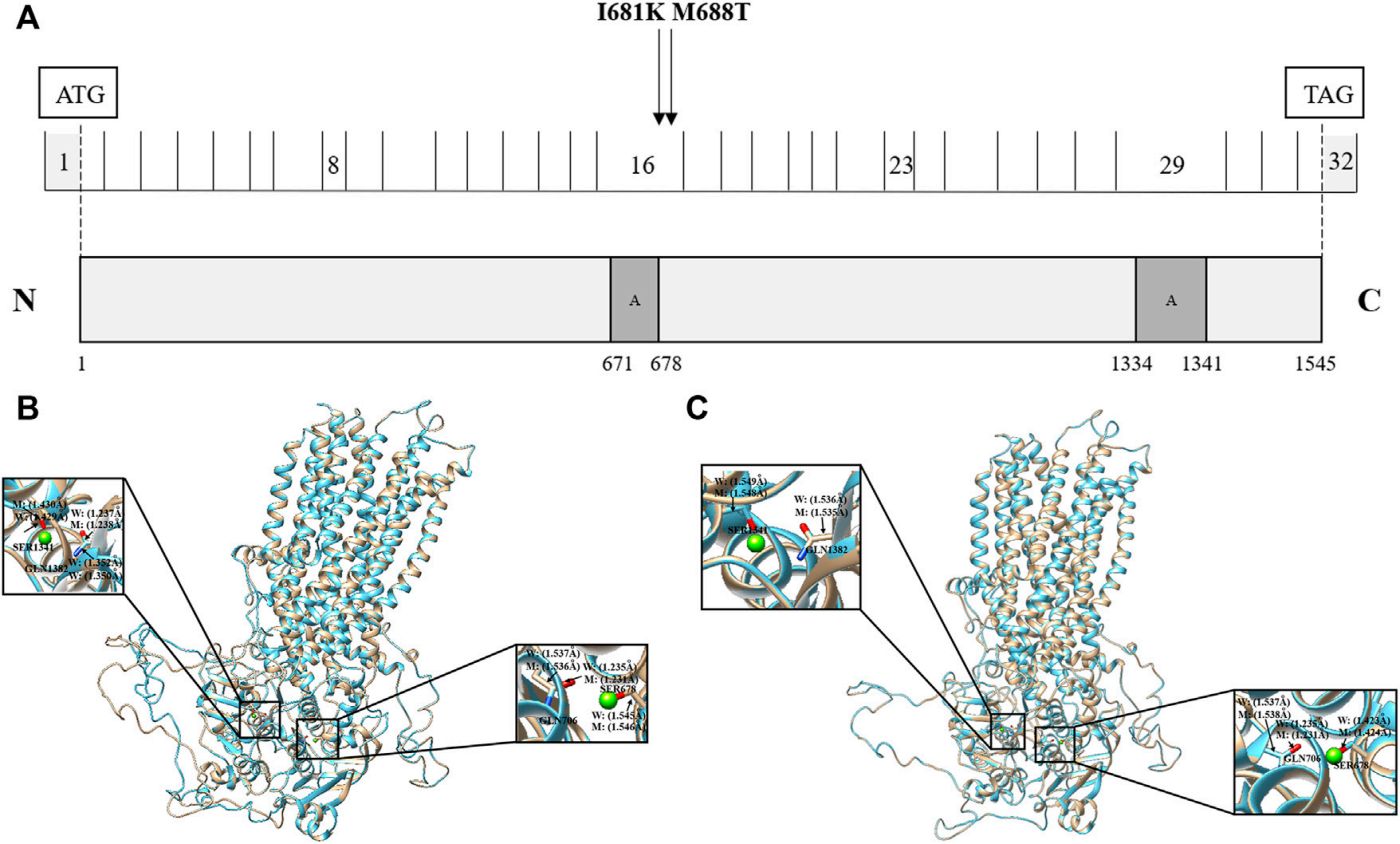
Effects of *ABCC2* Ile681Lys and Met688Thr variants on the protein sequence and structure. **(A)** Distribution of *ABCC2* variants. *ABCC2* is a 1545-amino acid protein containing two ATP binding cassette domains. Schematic representation of *ABCC2* NM_000,392.4 cDNA and protein showing the locations of the two novel possible pathogenic variants, Ile681Lys and Met688Thr, detected in two out of 249 ICP patients. Effects of *ABCC2* Ile681Lys **(B)** and Met688Thr variants **(C)** on the protein structure. The three-dimensional models of reference and modified (Ile681Lys and Met688Thr) ABCC2 are shown in gold and blue rounded structures, respectively. The enlarged portion shows that the two ATP binding cassette regions have small changes in the chemical bond lengths. **(A)** The ATP binding cassette region.

The *ABCC2* gene encodes a member of the superfamily of ATP-binding cassette transporters, which bind and hydrolyze ATP to enable the active transport of various substrates, including many drugs, toxicants, and endogenous compounds (bile acid, bile salt, bilirubin, etc.), across the cell membrane (Kamisako et al., 1999; Hayashi et al., 2005). ABCC2 has two ATP binding cassette domains, ATP 1 and ATP 2, which are located at positions 671–678 and 1,334–1,341, respectively (Figure 4A). In the present study, we identified two novel missense mutations at positions 681 (Ile681Lys) and 688 (Met688Thr). These two mutations have been predicted to be damaging to protein function according to the prediction results of SIFT and PolyPhen2 software.

To further investigate the possible effect of these two mutations on the ABCC2 protein structure, the reference and modified protein structures (Ile681Lys and Met688Thr) were compared simultaneously using UCSF Chimera 1.14rc. The results showed that the 3D model of the Ile681Lys and Met688Thr mutations both had a slight change in the chemical bond lengths of ATP-ligand binding amino acid side chains at positions Ser678, Gln706, Ser1342, and Gln1382 (Figure 4B, C).

### Correlations between the mutations and clinical data

In the mutation group, 15 pregnant women presented with preterm birth (32.61%, 15/46), and 7 pregnant women presented with MSAF (15.22%, 7/46). The descriptive statistics of 32 other clinical characteristics for patients with ICP with the 62 novel mutations are shown in Table 3. Regardless of whether the difference was significant, the mutation group tended to be associated with higher age and BMI and K, Na, Ca, P, Mg, WBC, RDW-SD, TBA, CHOL, TG, HDL, and LDL levels and lower gestational age and birth weight. It is worth noting that the mutation group (103.34 mmol/L) had a significantly (*p* = 0.04) lower Cl concentration than the wild-type (104.14 mmol/L) group. The associations between the clinical parameters (age: odds ratio (OR) = 1.008; 95%; confidence intervals (CI): 0.950–1.069; gestational age (OR = 0.991; 95% CI: 0.972–1.010); BMI (OR = 1.006, 95% CI: 0.919–1.102); gravidity (OR = 0.899, 95% CI: 0.726–1.114); parity (OR = 0.943, 95% CI: 0.636–1.398)) and the mutations were determined by logistic regression analysis.

**TABLE 3:** Descriptive statistics of 32 clinical features of ICP individuals associated with/without 62 novel mutations^a^.

	ICP with 62 novel mutations					ICP without 62 novel mutations					
Features	N	Mean	SD	Min.	Max.	N	Mean	SD	Min	Max	*P*
**Basic information**											
Age (years)	54	29.37	5.56	17.00	43.00	191	29.17	4.95	17.00	43.00	0.80
BMI (kg/m^2^)	52	25.85	3.64	17.08	36.20	187	25.78	3.31	18.90	38.50	0.89
Gestational age (days)	46	261.82	15.41	218	288	182	263.83	15.31	207	290	0.43
Gravidity (Times)	54	2.20	1.28	1	6	190	2.44	1.56	1	8	0.30
Parity (Times)	54	0.63	0.73	0	3	190	0.67	0.80	0	4	0.75
**Serum biochemical index**											
K (mmol/L)	56	4.05	0.44	3.40	6.40	193	3.98	0.31	3.20	4.90	0.26
Na (mmol/L)	56	137.29	2.02	133.00	143.00	193	137.26	2.31	132.00	145.00	0.95
Cl (mmol/L)	56	103.34	2.54	98.00	111.00	193	104.14	2.50	97.00	112.00	0.042^b^
Ca (mmol/L)	56	2.41	0.18	2.10	2.80	193	2.36	0.17	2.00	2.90	0.11
P (mmol/L)	56	1.20	0.22	0.80	1.70	193	1.17	0.21	0.70	1.80	0.40
Mg (mmol/L)	56	0.81	0.18	0.20	1.50	192	0.80	0.13	0.60	1.89	0.68
WBC (×10^9^)	56	8.84	2.31	4.11	15.50	193	8.50	2.80	4.37	24.23	0.40
RBC (×10^9^)	56	3.72	0.43	2.89	4.80	193	3.83	0.41	2.65	5.52	0.08
PLT (×10^9^)	56	190.21	65.28	81.00	412.00	193	200.45	61.17	75.00	476.00	0.28
RDW-SD (fL)	56	46.06	5.38	36.20	67.30	193	46.02	4.73	36.20	62.70	0.96
ALT (U/L)	56	89.77	112.68	3.00	506.00	193	103.12	129.84	4.00	595.00	0.49
AST (U/L)	56	74.54	90.85	13.00	509.00	193	87.76	98.13	12.00	456.00	0.37
GGT (U/L)	54	24.56	23.89	3.00	109.00	190	32.42	41.14	5.00	359.00	0.08
ALP (U/L)	54	166.81	81.60	39.00	399.00	190	175.81	78.59	43.00	487.00	0.46
TBA (μmol/L)	56	42.86	32.49	6.60	190.60	192	41.88	40.32	4.20	286.80	0.87
CG (mg/L)	39	11.05	10.57	0.70	42.70	159	11.06	14.84	0.30	88.60	0.99
TBIL (μmol/L)	54	13.17	5.29	6.20	30.30	191	14.97	8.19	5.30	67.90	0.06
DBIL (μmol/L)	54	6.06	4.23	2.00	25.20	191	6.97	6.53	0.90	52.50	0.23
IDBIL (μmol/L)	54	7.16	3.79	2.70	26.90	191	8.00	3.16	2.10	25.70	0.10
CHOL (mmol/L)	54	6.40	1.83	3.16	13.25	187	6.33	1.32	3.73	10.50	0.80
TG (mmol/L)	54	3.54	1.55	1.20	8.65	187	3.51	1.49	1.25	11.10	0.89
HDL (mmol/L)	54	2.08	0.68	1.08	5.34	187	1.93	0.46	0.92	4.06	0.14
LDL (mmol/L)	54	3.10	1.63	0.04	7.34	187	2.93	1.22	0.13	6.28	0.47
UA (μmol/L)	56	328.98	101.66	195.00	701.00	190	334.67	92.52	111.00	658.00	0.69
**Outcomes of pregnant women and newborns**											
Birth weight (kg)	46	2.93	0.52	1.40	3.90	179	3.06	0.57	1.23	5.30	0.17
Apgar score (1–10)	45	9.42	0.62	8	10	174	9.34	0.70	6	10	0.50
Bleeding amount (ml)	46	251.63	78.84	80	500.00	175	266.17	98.73	90.00	810.00	0.36

a See the footnotes in Table 1.

b Significant differences were underlined.

**FIGURE 5:**
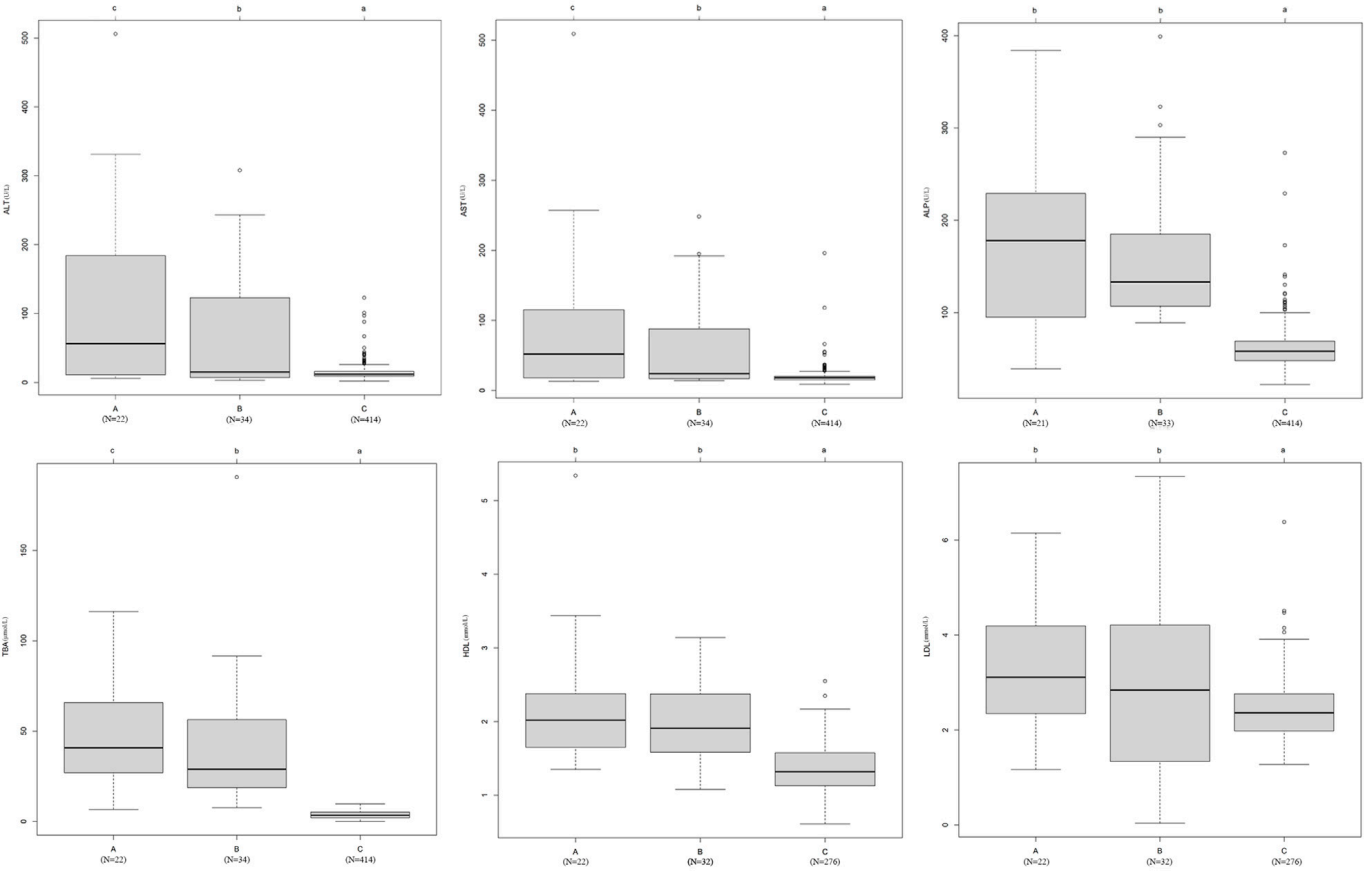
Effects of bile acid transporter and receptor gene mutations on biochemical indices. The difference in the average levels of ALT, AST, ALP, TBA, HDL, and LDL in the following groups: **(A)** 24 novel mutations in the damaging group, **(B)** 38 novel mutations in the probably damaging group, and **(C)** healthy control individuals without ICP disease. The means of different groups in rows with different superscript letters are significantly (*p* < 0.05) different from each other.

In addition, we found that patients with the 24 mutations in the damaging group (A) had higher ALT, AST, ALP, TBA, HDL, and LDL levels than patients with mutations in the probably damaging group (B) and the 414 local control individuals without ICP (C) (Figure 5). In particular, the levels of ALT, AST, and TBA were significantly different among the three groups (*p* < 0.05).

## Discussion

To the best of our knowledge, this study is the largest analysis to use WES technology to identify potential novel possibly pathogenic mutations in OTAP genes, ABC genes, and receptor family genes involved in bile acid transporters and receptors. We identified 62 novel mutations in 82 genes in 249 ICP patients.

The present study has 3 major strengths. First, many previous studies that have identified the genetic loci contributing to ICP disease were mainly conducted by sequencing a single gene in a small number of individuals or a disease-affected family. This can omit some potentially pathogenic mutations. Recently, WES technology has proved to be a powerful new approach for detecting low-frequency (0.01 ≤ MAF <0.05) and rare (MAF <0.01) variations that affect human diseases, such as spontaneous preterm birth (Huusko et al., 2018) and fetal hydrocephalus (Sun et al., 2019). Using this method, we have also successfully revealed *ANO8* as a genetic risk factor for ICP (Liu et al., 2020) and identified 42 novel mutations in ABC transporter genes that are associated with ICP in 151 samples (Liu et al., 2021). Moreover, by using WES, our present study also successfully identified novel candidate potential disease-causing loci in 82 genes that are currently known to have functions related to bile acid transporters and receptors. Second, this is the first systematic identification of OTAP, ABC, and receptor gene mutations from a relatively large sample of ICP patients in China (n = 249). Third, we collected relatively complete clinical data from these patients to support the potential association between mutations and clinical data and to provide further insight into the mechanisms of ICP disease. Certainly, our study also has limitations. First, most of the patient samples were collected from Jiangxi Province, which may limit the applicability of the generality of these results. However, this study is still meaningful because the incidence of ICP in Jiangxi reached as high as 3–5%. In addition, in this study, we only collected a simple sample of subjects for WES. However, genotypic information from parents provided by trio-based WES enabled the detection of a high percentage of *de novo* variants inside the ICP disease cohort. However, considering the number (249) of ICP samples, the focus of the analysis was extended beyond well-known ICP-related genes and bioinformatics analyses, thus making it possible to find new candidates. In addition, this study identified some interesting novel pathogenic loci that were associated with ICP; however, the causality between loci and ICP disease needs to be verified by further functional experiments.

The OTAP family is a type of bidirectional transport family of multispecific membrane carriers that can transport many types of endogenous and exogenous substances, such as bile acids and steroid hormones. This implies that OTAP abnormalities can affect bile acid levels (Dawson et al., 2005). Previous studies have focused more on the effect of the expression of OTAPs, such as *SLC10A2* and *SLCO4A1*, on maternal bile acid levels (Yan et al., 2015; Ontsouka et al., 2021). Our present study identified 13 new mutations, including *SLC10A2* and Ala310Thr, in the OTAP family from 249 ICP patients. This result extends the role of OTAPs in ICP. These mutations affect a specific pathway/mechanism of ICP and need further experimental verification.

Compared to solute carriers and receptors, most of the reports about the genetic susceptibility to ICP have thus far mainly focused on the ABC gene series. Since the *ABCB4* gene mutation was first reported to be associated with ICP in a Caucasian population in 1999, more heterozygous mutant alleles of functionally known genes, including *ABCB4, ABCB11, ABCC2, ATP8B1,* and *TJP2*, have been described (Jacquemin et al., 1999; Bacq et al., 2009; Dixon et al., 2014; Dixon et al., 2017; Piatek et al., 2018; Aydin et al., 2020). In this study, we extended the analysis across all ABC genes and revealed genetic mutations, including *ABCB4* Ile377Asn, Ser1153Asn and Asp972Asn, *ABCB11* Ala588Pro, *ABCC2* Ile681Lys, Met688Thr, and Ile487Val. Among them, the 3D model construction analysis indicated that the *ABCC2* Ile681Lys and Met688Thr mutations altered the amino side chains. This change could affect the binding efficiency of the ATP molecule and further change the transport function. This effect is similar to the effect of the *ABCC2* Ser1342Tyr mutation that we previously identified (Liu et al., 2021). Consistent with this result, Corpechot et al. 2020 reported the genetic contribution of *ABCC2* to inherited cholestatic disorders, indicating that *ABCC2* is more closely related to ICP disease. The results of this study not only confirmed the functionally known genes but also expanded the role of new mutations in other ABC genes. This deepens our understanding of the pathogenesis of ABC genes in ICP.

In recent years, an accumulating body of evidence has demonstrated that nuclear receptors are key regulators of various processes, including the metabolism of xeno- and endobiotics such as bile acids and drugs (Ahmad and Haeusler, 2019). These receptors are generally considered to be therapeutic targets for cholestatic liver disease (Van Mil et al., 2007; Manna and Williamson, 2021). Recent studies have also made significant progress in identifying the genetic mutations that contribute to ICP disease. For example, functional variants in *FXR* were implicated as a genetic predisposition for ICP in a European population (Van Mil et al., 2007; Zimmer et al., 2009). Additionally, we also found two missense mutations (Ser145Phe and Met185Leu) that were implicated in individual susceptibility to ICP disease (Lai et al., 2022). Our present study detected two novel mutations, including the nonsense mutation Tyr149Ter and the missense mutation Glu199Gly, which might contribute to the development of ICP. Of course, the mechanisms that affect ICP need to be further validated. In addition to *FXR*, the common gene variants of *PXR* have also demonstrated genetic susceptibility to ICP. Castano et al. 2010 identified that *PXR* polymorphisms (rs2461823 G allele) are significantly associated with ICP and adverse pregnancy outcomes, including low birth weight and Apgar score, by comparing a total of 101 ICP patients and 171 control individuals. In this study, two missense mutations (Gln253Lys and Gln465Arg) were identified in the *PXR* gene. Furthermore, we also first identified other receptor genes with novel mutations that are susceptible to ICP. These results expand the receptor gene mutation datasets and provide more available targets for the treatment of ICP. To our knowledge, this is the first study in which whole-exome sequencing has been performed to systematically identify mutations in the receptor genes in ICP.

In recent decades, bile acid signaling molecules have been shown to activate bile acid receptors in the nucleus and membrane of cells, thereby regulating lipids, glucose, and metabolism in the liver (Lefebvre et al., 2009; Chiang, 2013; Ahmad and Haeusler, 2019). This implies that the accumulation of TBA might lead to lipid abnormalities. Consistent with this result, our results showed that the mutation group had higher CHOL, TG, HDL, and LDL levels than the wild-type group (Table 3). The average levels of HDL and LDL were higher in the group with the 24 damaging mutations than in the group with the 38 probably damaging mutations and the healthy group with no mutations (Figure 5). This finding suggests that the effect size of the mutations of the damaging group was higher than that of the probably damaging group for the lipid indices.

## Conclusion

In conclusion, this is the first study to conduct WES to reveal the genetic variants in bile acid transporters and receptor genes that are associated with ICP disease. We identified 62 novel potentially pathogenic mutations in 82 genes in 249 ICP patients. In particular, out of the 62 novel mutations, 24 were classified as damaging. Functional validation and experimental verification of these 62 novel mutations need to be further investigated. Our findings provide new insights into the genetic basis of ICP disease and suggest potential candidate variants for ICP clinical treatment.

## Data Availability

The datasets presented in this study can be found in online repositories. The names of the repository/repositories and accession number(s) can be found in the article/Supplementary Material.
