# Supplementary material for: Whole-exome sequencing expands the roles of novel mutations of organic anion transporting polypeptide, ATP-binding cassette transporter, and receptor genes in intrahepatic cholestasis of pregnancy
Source: Front Genet. 2022 Aug 15;13:941027. doi: 10.3389/fgene.2022.941027 (PMC9421141; doi:10.3389/fgene.2022.941027)
Supplement: Supplementary file 2 [file Table1.DOCX]

**Supplementary Table S1.** Primes used to confirm the variants in the genes.

| **Order** | **Gene** | **Patient** | **Annealing temperature (℃)** | **Amplicon (bp)** | **Forward primer (5’-3’)** | **Reverse primer (5’-3’)** |
| --- | --- | --- | --- | --- | --- | --- |
| 1 | *SLCO1A2* | ICP187 | 56 | 232 | TGCCCACATTGTCCTCATTA | CCCCTCAGCAGAAAGTGTTT |
| 2 | *SLCO1B1* | ICP31 | 56 | 167 | TAATGGGGCCATTCAACTGT | GGAAACCCAGTGCAAGTGAT |
| 3 | *SLC12A3* | ICP258 | 56 | 207 | AGGCCATAGACGTGGTGAAG | CTTCCCAGAGCTGAGACACC |
| 4 | *ABCA2* | ICP212 | 55 | 194 | TGGAGGAACACCTCTGGTTC | TTTGTTGGAGAGCTCCAGGT |
| 5 | *ABCA3* | ICP177 | 56 | 168 | AGAAGACAGTGACCCCGAGA | ACTGCCGTGCTGGTAAGTCT |
| 6 | *ABCA10* | ICP220 | 56 | 174 | AGCACCATCCAGATGACCTC | TCCTGTGGGAGAAGTTTCCA |
| 7 | *ABCB4* | ICP192 | 50 | 157 | CATTCCAGGTCCTATTTTTGGA | CCCTTTGATGCTGTCTGGTT |
| 8 | *ABCB11* | ICP236 | 56 | 181 | GTGGCCAGAAACAAAGGGTA | CAGCACAAGCATTTCCACAT |
| 9 | *ABCC6* | ICP260 | 56 | 236 | GGCTCATCCTTCCTCACAGA | GATGGGGACATCCTAGCAGA |
| 10 | *ABCC11* | ICP256 | 56 | 160 | TTCTCTCATGGTCTGCGTTG | AGGACCAGGAACTGCTCTGA |
| 11 | *CHRM3* | ICP245 | 56 | 237 | TTTGGAATCTGGGCTACTGG | GTGTGCGTTTTGTCACTGCT |
| 12 | *NR1H4* | ICP270 | 56 | 173 | ATCAAAGGGGATGAGCTGTG | TGGGAAAAGGGAAGCAATTA |
