# Supplementary figures and images for: Whole-exome sequencing expands the roles of novel mutations of organic anion transporting polypeptide, ATP-binding cassette transporter, and receptor genes in intrahepatic cholestasis of pregnancy
Source: Front Genet. 2022 Aug 15;13:941027. doi: 10.3389/fgene.2022.941027 (PMC9421141; doi:10.3389/fgene.2022.941027)

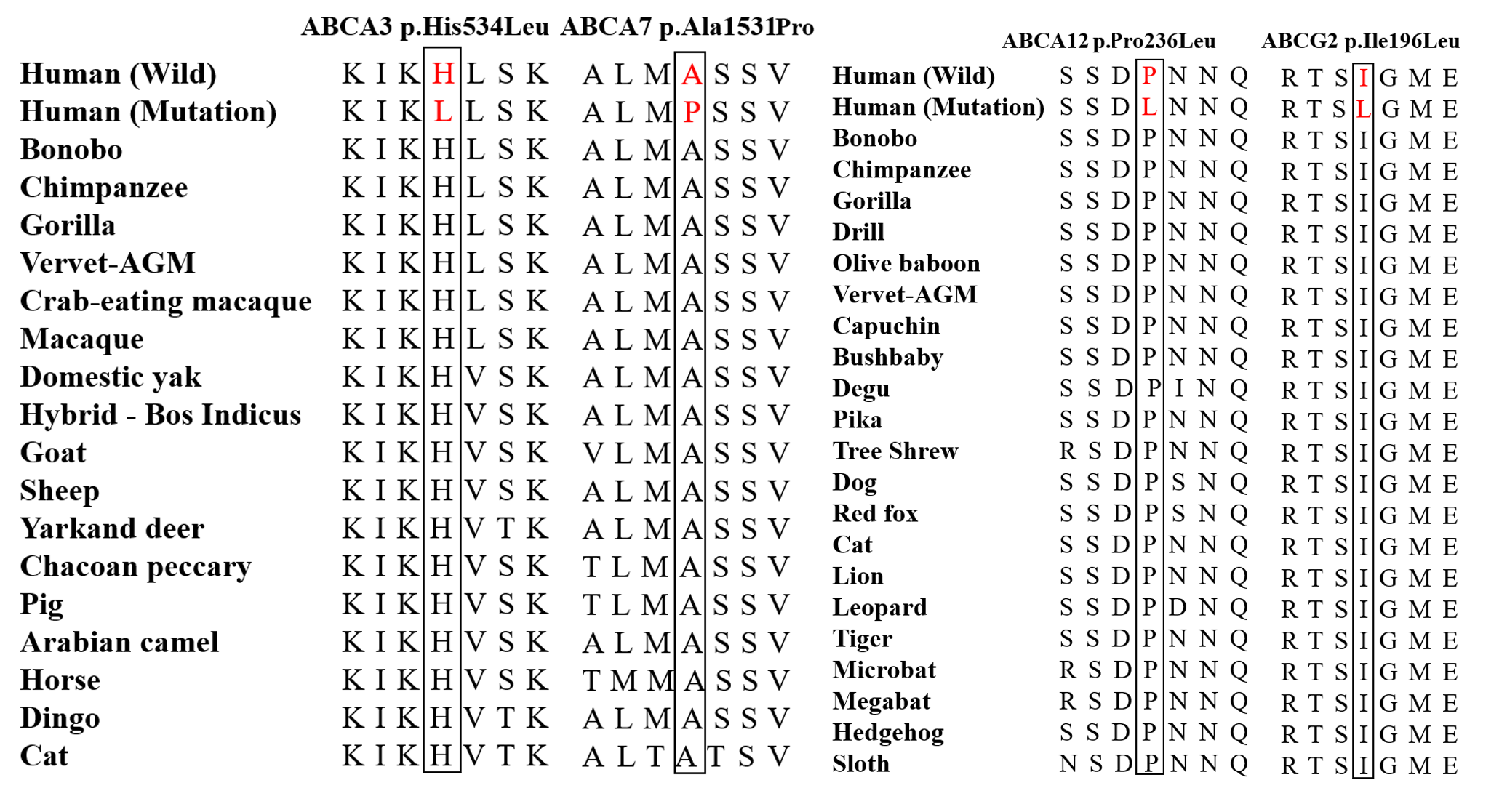

Supplement: Supplementary file 3 [file Image1.TIF]
