# Supplementary material for: Whole-exome sequencing expands the roles of novel mutations of organic anion transporting polypeptide, ATP-binding cassette transporter, and receptor genes in intrahepatic cholestasis of pregnancy
Source: Front Genet. 2022 Aug 15;13:941027. doi: 10.3389/fgene.2022.941027 (PMC9421141; doi:10.3389/fgene.2022.941027)
Supplement: Supplementary file 4 [file Table2.DOCX]

**Supplementary Table S2.** Overview of currently known genes associated with bile acids transports and receptors.

| **Membrane Protein Class** | **Gene Name** | **Previous Symbols/Aliases** | **Approved Name** |
| --- | --- | --- | --- |
| **Solute carriers** | *SLC10A1* | NTCP | Solute carrier family 10 member 1 |
|  | *SLC10A2* | ASBT/ISBT | Solute carrier family 10 member 2 |
|  | *SLC12A3* | NCCT | Solute carrier family 12 member 3 |
|  | *SLC51A* | OSTalpha | Solute carrier family 51 subunit alpha |
|  | *SLC51B* | OSTbeta | Solute carrier family 51 subunit beta |
|  | *SLCO1A2* | SLC21A3/OATP1A2 | Solute carrier organic anion transporter family member 1A2 |
|  | *SLCO1B1* | SLC21A6/OATP1B1 | Solute carrier organic anion transporter family member 1B1 |
|  | *SLCO1B3* | SLC21A8/OATP1B3 | Solute carrier organic anion transporter family member 1B3 |
|  | *SLCO1C1* | SLC21A14/OATP1C1 | Solute carrier organic anion transporter family member 1C1 |
|  | *SLCO2A1* | SLC21A2/OATP2A1 | Solute carrier organic anion transporter family member 2A1 |
|  | *SLCO3A1* | SLC21A11/OATP3A1 | Solute carrier organic anion transporter family member 3A1 |
|  | *SLCO4A1* | SLC21A12/OATP4A1 | Solute carrier organic anion transporter family member 4A1 |
|  | *SLCO5A1* | SLC21A15/OATP5A1 | Solute carrier organic anion transporter family member 5A1 |
|  | *SLCO6A1* | OATPY/OATP6A1 | Solute carrier organic anion transporter family member 6A1 |
|  | *SLCO2B1* | SLC21A9/OATP2B1 | Solute carrier organic anion transporter family member 2B1 |
|  | *SLCO4C1* | SLC21A20/OATP4C1 | Solute carrier organic anion transporter family member 4C1 |
| **ABC transporters** | *ABCA1* | ABC1 | ATP binding cassette subfamily A member 1 |
|  | *ABCA2* | ABC2 | ATP binding cassette subfamily A member 2 |
|  | *ABCA3* | ABC3 | ATP binding cassette subfamily A member 3 |
|  | *ABCA4* | ABCR | ATP binding cassette subfamily A member 4 |
|  | *ABCA5* | EST90625 | ATP binding cassette subfamily A member 5 |
|  | *ABCA6* | EST155051 | ATP binding cassette subfamily A member 6 |
|  | *ABCA7* | ABCX | ATP binding cassette subfamily A member 7 |
|  | *ABCA8* | KIAA0822 | ATP binding cassette subfamily A member 8 |
|  | *ABCA9* | EST640918 | ATP binding cassette subfamily A member 9 |
|  | *ABCA10* | EST698739 | ATP binding cassette subfamily A member 10 |
|  | *ABCA12* | ICR2B | ATP binding cassette subfamily A member 12 |
|  | *ABCA13* | FLJ33876 | ATP binding cassette subfamily A member 13 |
|  | *ABCB1* | MDR1 | ATP binding cassette subfamily B member 1 |
|  | *ABCB4* | MDR2/MDR3 | ATP binding cassette subfamily B member 4 |
|  | *ABCB5* | EST422562 | ATP binding cassette subfamily B member 5 |
|  | *ABCB6* | EST45597 | ATP binding cassette subfamily B member 6 |
|  | *ABCB7* | ABC7 | ATP binding cassette subfamily B member 7 |
|  | *ABCB8* | EST328128 | ATP binding cassette subfamily B member 8 |
|  | *ABCB9* | EST122234 | ATP binding cassette subfamily B member 9 |
|  | *ABCB10* | EST20237 | ATP binding cassette subfamily B member 10 |
|  | *ABCB11* | BSEP/PFIC2 | ATP binding cassette subfamily B member 11 |
|  | *ABCC1* | MRP/MRP1 | ATP binding cassette subfamily C member 1 |
|  | *ABCC2* | MRP2 | ATP binding cassette subfamily C member 2 |
|  | *ABCC3* | MRP3 | ATP binding cassette subfamily C member 3 |
|  | *ABCC4* | MRP4 | ATP binding cassette subfamily C member 4 |
|  | *ABCC5* | MRP5 | ATP binding cassette subfamily C member 5 |
|  | *ABCC6* | MRP6 | ATP binding cassette subfamily C member 6 |
|  | *ABCC8* | MRP8 | ATP binding cassette subfamily C member 8 |
|  | *ABCC9* | SUR2 | ATP binding cassette subfamily C member 9 |
|  | *ABCC10* | MRP7 | ATP binding cassette subfamily C member 10 |
|  | *ABCC11* | MRP8 | ATP binding cassette subfamily C member 11 |
|  | *ABCC12* | MRP9 | ATP binding cassette subfamily C member 12 |
|  | *ABCD1* | ALD | ATP binding cassette subfamily D member 1 |
|  | *ABCD2* | ALDL1 | ATP binding cassette subfamily D member 2 |
|  | *ABCD3* | PXMP1 | ATP binding cassette subfamily D member 3 |
|  | *ABCD4* | PXMP1L | ATP binding cassette subfamily D member 4 |
|  | *ABCE1* | OABP | ATP binding cassette subfamily E member 1 |
|  | *ABCF2* | ABC28 | ATP binding cassette subfamily F member 2 |
|  | *ABCF3* | EST201864 | ATP binding cassette subfamily F member 3 |
|  | *ABCG1* | ABC8 | ATP binding cassette subfamily G member 1 |
|  | *ABCG2* | BCRP | ATP binding cassette subfamily G member 2 |
|  | *ABCG4* | WHITE2 | ATP binding cassette subfamily G member 4 |
|  | *ABCG5* | STSL | ATP binding cassette subfamily G member 5 |
|  | *ABCG8* | GBD4 | ATP binding cassette subfamily G member 8 |
| **Receptors** | *AHR* | BHLHe76 | Aryl hydrocarbon receptor |
|  | *CHRM1* | HM1 | Cholinergic receptor muscarinic 1 |
|  | *CHRM2* | HM2 | Cholinergic receptor muscarinic 2 |
|  | *CHRM3* | HM3 | Cholinergic receptor muscarinic 3 |
|  | *CHRM4* | HM4 | Cholinergic receptor muscarinic 4 |
|  | *CHRM5* | HM5 | Cholinergic receptor muscarinic 5 |
|  | *GPBAR1* | TGR5 | G protein-coupled bile acid receptor 1 |
|  | *NR1H2* | LXRb | Nuclear receptor subfamily 1 group H member 2 |
|  | *NR1H3* | LXRa | Nuclear receptor subfamily 1 group H member 3 |
|  | *NR1H4* | FXR | Nuclear receptor subfamily 1 group H member 4 |
|  | *NR1I2* | PXR | Nuclear receptor subfamily 1 group I member 2 |
|  | *NR1I3* | CAR | Nuclear receptor subfamily 1 group I member 3 |
|  | *NR0B2* | SHP | Nuclear receptor subfamily 0 group B member 2 |
|  | *PPARA* | PPAR | Peroxisome proliferator activated receptor alpha |
|  | *PPARD* | NR1C2 | Peroxisome proliferator activated receptor delta |
|  | *PPARG* | NR1C3 | Peroxisome proliferator activated receptor gamma |
|  | *RXRA* | NR2B1 | Retinoid X receptor alpha |
|  | *RXRG* | NR2B3 | Retinoid X receptor gamma |
|  | *S1PR2* | Gpcr13 | Sphingosine-1-phosphate receptor 2 |
|  | *VDR* | NR1I1 | Vitamin D receptor |
| **Others** | *ATP8B1* | FIC1/BRIC/PFIC1 | ATPase phospholipid transporting 8B1 |
|  | *TJP2* | ZO2 | Tight junction protein 2 |
